# Supplementary material for: From lab to real life: Is there a link between lab-based and ecological assessment of Procedural Perceptual-Motor Learning tasks?
Source: PLoS One. 2025 Apr 7;20(4):e0319715. doi: 10.1371/journal.pone.0319715 (PMC11975140; doi:10.1371/journal.pone.0319715)
Supplement: S1 Table — (DOCX) [file pone.0319715.s001.docx]

**Scoring accuracy in tasks measuring SL**

The scoring for accuracy is the same for all tasks measuring SL: 0.5 points are awarded if the element is correctly executed and 0.5 points are awarded if the element is placed correctly (following the correct preceding element).

**SL_1: Learning a sign language word**

**
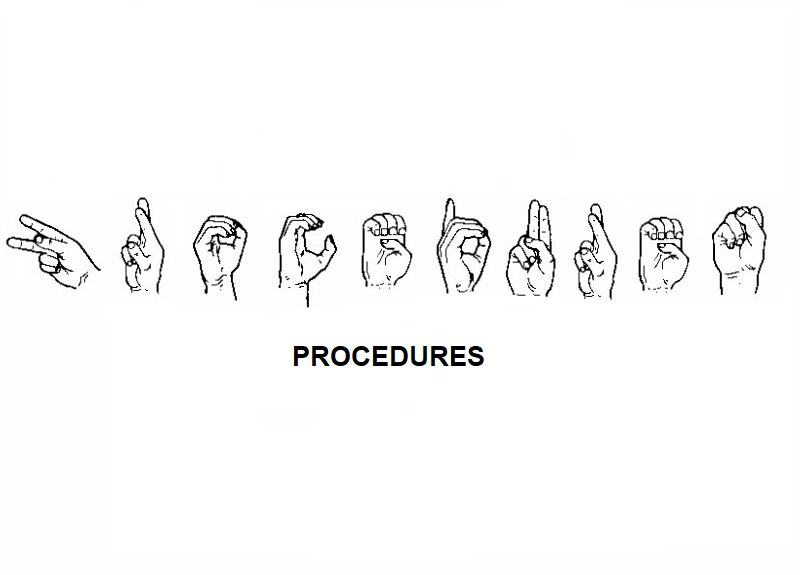
**

| **Sequence** | **Correct execution** | **Trial 1** | **Trial 2** | **Trial…** |
| --- | --- | --- | --- | --- |
| 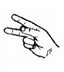 | The middle and index fingers are extended horizontally and spread apart. The thumb holds the ring and little fingers. |  |  |  |
| 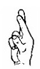 | Palm is outward.  Middle and index fingers are crossed. |  |  |  |
| 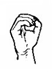 | All fingers are flexed.  The thumb touches one of the fingers. |  |  |  |
| 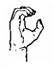 | All fingers are flexed.  No thumb contacts. |  |  |  |
| 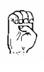 | Palm is outward.  All fingers flexed (not closed fist). |  |  |  |
| 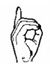 | Index finger is extended.  The other fingers are bent and the thumb is in contact with one of the fingers. |  |  |  |
| 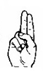 | Palm is outward.  Index and middle fingers are extended |  |  |  |
| 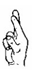 | Palm is outward.  Middle and index fingers are crossed. |  |  |  |
| 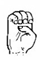 | Palm is outward.  All fingers flexed (not closed fist). |  |  |  |
| 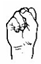 | Palm is outward. The point is closed with the thumb. |  |  |  |

**SL_2: Learning to tie a knot**


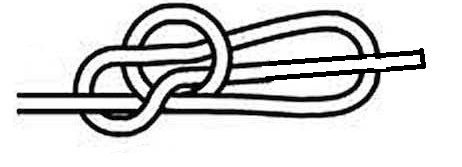


| **Sequence** | **Correct execution** | **Trial 1** | **Trial 2** | **Trial…** |
| --- | --- | --- | --- | --- |
| 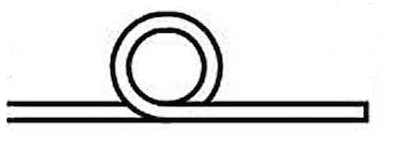 | Make the loop |  |  |  |
| 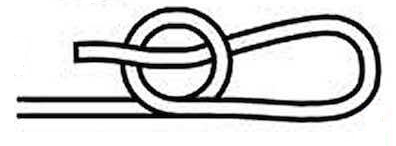 | Feed the rope into the loop from below |  |  |  |
| 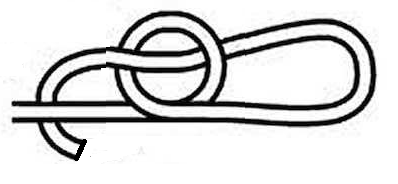 | The 2 strands must cross (the one coming from the loop passes under the other). |  |  |  |
| 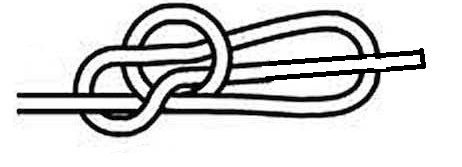 | The strand returns to the first loop and emerges in the 2nd. |  |  |  |

**SL_3: Learning a musical staff sequence:**


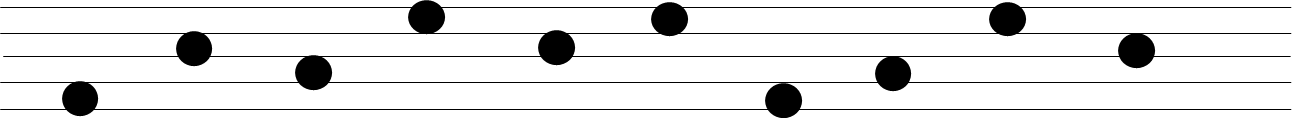


| **Sequence** | **Correct execution (key pressed)** | **Trial 1** | **Trial 2** | **Trial…** |
| --- | --- | --- | --- | --- |
| **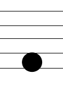** | **C** |  |  |  |
| 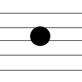 | **B** |  |  |  |
| **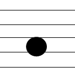** | **V** |  |  |  |
| **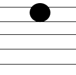** | **N** |  |  |  |
| 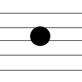 | **B** |  |  |  |
| **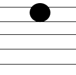** | **N** |  |  |  |
| **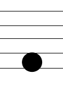** | **C** |  |  |  |
| **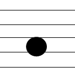** | **V** |  |  |  |
| **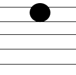** | **N** |  |  |  |
| 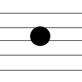 | **B** |  |  |  |
