# Supplementary material for: From lab to real life: Is there a link between lab-based and ecological assessment of Procedural Perceptual-Motor Learning tasks?
Source: PLoS One. 2025 Apr 7;20(4):e0319715. doi: 10.1371/journal.pone.0319715 (PMC11975140; doi:10.1371/journal.pone.0319715)
Supplement: Attachment — Submitted filename: Response to reviewers.docx [file pone.0319715.s003.docx]

**Comments to the Author**

1. Is the manuscript technically sound, and do the data support the conclusions?

The manuscript must describe a technically sound piece of scientific research with data that supports the conclusions. Experiments must have been conducted rigorously, with appropriate controls, replication, and sample sizes. The conclusions must be drawn appropriately based on the data presented.

Reviewer #1: Partly

Reviewer #2: Yes

2. Has the statistical analysis been performed appropriately and rigorously?

Reviewer #1: Yes

Reviewer #2: Yes

3. Have the authors made all data underlying the findings in their manuscript fully available?

The [PLOS Data policy](http://c05y1x9s.r.us-east-2.awstrack.me/L0/http:%2F%2Fwww.plosone.org%2Fstatic%2Fpolicies.action%23sharing/1/010f0191ba8a5740-a1b30736-8268-43e8-a18e-326055cf9d18-000000/d-O-TWju_r34n2JI41TphqU_Obg=175) requires authors to make all data underlying the findings described in their manuscript fully available without restriction, with rare exception (please refer to the Data Availability Statement in the manuscript PDF file). The data should be provided as part of the manuscript or its supporting information, or deposited to a public repository. For example, in addition to summary statistics, the data points behind means, medians and variance measures should be available. If there are restrictions on publicly sharing data—e.g. participant privacy or use of data from a third party—those must be specified.

Reviewer #1: Yes

Reviewer #2: Yes

4. Is the manuscript presented in an intelligible fashion and written in standard English?

PLOS ONE does not copyedit accepted manuscripts, so the language in submitted articles must be clear, correct, and unambiguous. Any typographical or grammatical errors should be corrected at revision, so please note any specific errors here.

Reviewer #1: Yes

Reviewer #2: Yes

5. Review Comments to the Author

Please use the space provided to explain your answers to the questions above. You may also include additional comments for the author, including concerns about dual publication, research ethics, or publication ethics. (Please upload your review as an attachment if it exceeds 20,000 characters)

Reviewer #1: This study asks whether the results of experiments conducted in a controlled laboratory environment on motor skill learning can be used to predict and understand how these skills are performed in real-life everyday situations.
Answering this question is crucial for researchers and specialists working in the field of motor learning. If we can establish a link between laboratory findings and real-world performance, we can: Gain a better understanding of the motor learning process, design more effective training methods, help individuals improve their performance in various activities.
These researchers aimed to determine whether the results of simple laboratory experiments could predict an individual's performance in everyday tasks. For instance, they investigated whether a person who can effectively learn a motor sequence in a laboratory setting can also apply this skill proficiently in real-world scenarios. Their findings suggest that there is indeed a partial correlation between laboratory performance and real-world abilities. These results hold significant implications for rehabilitation professionals. This can be good, however, there are several concerns about this manuscript:

1- The introduction is long and story-like and suitable for textbooks and not scientific articles.

We have revised the introduction to better align with the expectations of a scientific article.

2- To measure motor sequence learning, it did not follow the method provided by Nissen and Bullemer and self-made assignment is used.

The reviewer is right in the sense that our method to measure SL is adapted from the method provided by Nissen and Bullemer. For greater clarity, we added line 186: “*we adapted the task initially introduced by Nissen and Bullemer (1987). Similar to their method, the stimuli appeared in four different locations. However, instead of responding by pressing a corresponding key, participants were required to use the mouse to click directly on the stimulus.* *The regular patterns within the sequence are acquired in a manner similar to how participants interact with a keyboard or mouse (Chambaron et al., 2008*)”.

3- Figure 1 is completely unclear and ambiguous in terms of quality, and it is not possible to evaluate it. This problem exists in other shapes and images as well.

We are sorry about this point but the cause seems to be the conversion to pdf in the submission process. Indeed, the original figures which have been submitted was 2246 wide for 2446 high with a 300 dpi resolution (which perfectly answers to the submission's artwork guidelines).

We regret that the compiled submission PDF includes low-resolution preview images of the figures. We submit the revised version of our manuscript with the figures in high resolution, hoping that they will be more legible.

It is possible that the document you received includes a link at the top of the page that allows you to view the image in .tif format.

4- It is not clear why the general health questionnaires and Mini Mental State Examination were not used to evaluate the participants, and only self-reports were used to evaluate the participants.

Thank you for your comment regarding the evaluation of participants. We appreciate the importance of thorough assessment in research. However, we did not find it necessary to administer the Mini-Mental State Examination (MMSE) for our younger adult population, as the prevalence of significant cognitive impairment is generally low in this age group. Our primary goal was to balance the need for controlling variables with the feasibility of the study, particularly regarding time constraints for participants. Given these considerations, we opted to rely on self-reports and targeted inclusion criteria to ensure a sample free of neurological or psychiatric diagnoses. While we recognize that general health questionnaires and more extensive cognitive assessments could provide additional insights, we believe our approach adequately addressed the objectives of the study without overburdening participants or complicating the research process.

Furthermore, our inclusion criteria were carefully structured to recruit individuals without a history of neurological or psychiatric conditions that could impact the results. This approach aimed to ensure that our sample was as homogenous as possible concerning cognitive health. While we recognize the value of comprehensive health assessments, we believe our methodology, given the study's specific focus and constraints, was appropriate for the research objectives.

5- Number of participants: The sample size of 42 participants is relatively small, especially considering the number of variables being examined. This could limit the statistical power of the study.

We acknowledge that this relatively small sample size may limit the statistical power of our study., particularly given the number of variables being examined

We acknowledge that this relatively small sample size may limit the statistical power of our study, particularly given the number of variables being examined. We added line 604: “*The limited sample size constrains the robustness of the conclusions and reduces the statistical power of the analysis, warranting careful interpretation of the results*.”

6- Gender imbalance: Out of 42 participants, 31 were women. This imbalance could affect the generalizability of the results.

We appreciate your concern about the gender imbalance in our sample. We recognize that this imbalance may affect the generalizability of our findings. A quick review of the literature on the effect of gender on PPML reveals that this issue has been scarcely studied. For SA, no significant differences have been found (<https://pubmed.ncbi.nlm.nih.gov/21182785/>), or only among older participants (https://pubmed.ncbi.nlm.nih.gov/36617621/). For SL, we did not find any studies specifically addressing this question.

We have added a statement in the manuscript (line 607): “*Even if, as far as we know, gender differences are not reported in the literature, it should be noted that our sample~~,~~ is not strictly representative of the general population*”.

7. Definition of "ecological": Although the "ecological" tasks are designed to reflect real-life conditions, they are still performed in a controlled laboratory environment. This might call into question their true "ecological" nature.

Our tasks are specifically designed to replicate real-life situations and contexts, allowing for the observation of skill acquisition in conditions that closely resemble those encountered in everyday life. Although the controlled environment may limit certain aspects of realism, the behaviors and tools utilized in these tasks are reflective of participants' daily experiences. Moreover, similar studies in the field have successfully employed laboratory settings to examine real-life behaviors (references 14 to 27), demonstrating that valuable insights can still be gained. We believe that the relevance of our findings to real-world applications justifies our use of the term “ecological”.

8. Task complexity: Some of the "ecological" tasks (such as learning sign language) may be more complex than the laboratory tasks, which could make direct comparison difficult.

We acknowledge that ecological tasks inherently involve more perceptual, cognitive, and motor factors than the laboratory tasks. However, despite these differences in task complexity, our hypothesis of correlation is driven by the shared learning processes.

As discussed in our manuscript (line 585), it is also possible that some correlations were not significant because of the task-specific complexity. This is the case for two tasks which do not correlate neither with SL not with SA: SL3: Learning a musical staff sequence, and SA_3: Walking without dropping a ball. As attested by the learning curves, learning process takes place for these tasks but it is possible that other cognitive or motor processes are pregnant than the procedural perceptive-motor learning process.

9. Task order: Although the ecological tasks were presented randomly, the laboratory tasks were always performed first. This could lead to an order effect.

Thank you for this comment. The laboratory tasks were intentionally performed first to establish a controlled and consistent baseline across participants. This approach allows us to minimize variability in performance due to familiarity with the tasks. In contrast, the ecological tasks involve materials and activities with which the participants are generally more familiar in their daily lives. Because they can directly perceive their success or failure in these tasks, this familiarity could have a stronger influence on their performance. By conducting the ecological tasks after the laboratory tasks, we aimed to prevent any potential carryover effects from this heightened sense of self-assessment and familiarity.

9. Criteria for selecting ecological tasks: More details about how the 8-person panel classified the tasks could have been useful.
These points do not necessarily indicate weaknesses in the study, but they are aspects that could be considered when interpreting the results and discussing the limitations of the study.

Thanks for this comment. We added clarifications in line 580: “*Our ecological tasks have been classified by eight researchers and clinicians as predominantly involving either SL or SA. However, this classification may not fully account for the possibility that some tasks may integrate aspects of both types of learning**. Our results suggest that this is the case given that our hypotheses are not fully validated”*.

10- The data portion of this study has significant strengths, including comprehensiveness of data, appropriate statistical methods, and transparency in reporting. However, the relatively small sample size and the need for more detail on the handling of missing data can be considered as weaknesses.

Thank you for this comment. Please see our response to comment 5 and line 604-606 for the answer.

Reviewer #2: First of all, I would like to say to the respected authors of this paper, don't get tired; This paper seems to be a valuable and good research. Nevertheless, it is recommended that the following points be considered in different sections of the paper by respected researchers.

Abstract:
It is recommended that this section should be in accordance with the format of the journal and should be added to the type of study in the methods section and inferential statistics report in the results section.

Thank you for your comment. It seems to us that the journal does not appear to impose a specific format for the abstract section. However, in line with your comment, we have added line 41 “*in an original research”* and line 42 “*with non-parametric repeated measures ANOVA, Spearman’s rank correlation tests were performed”*.

Introduction:
Considering that the current research is considered a practical and new study, it is expected that its necessity and importance will be expressed more clearly.

Thank you for this comment. We have added line 137: “*Although laboratory tasks have been widely used to study motor learning, their relevance to real-world skill acquisition remains debated. Ecological tasks provide a more realistic observation of how skills are acquired but their measurements require materials, time and careful quotation by trained assessors. Given the potential implications of evaluating skill learning before rehabilitation, teaching, and coaching, it is important to assess whether these two types of tasks are comparable*”.

Method:
It seems appropriate. It is recommended to make this section shorter and more concise if possible.

We understand your comment but it seems difficult to condense this section without compromising clarity and understanding. We have, however, reduced the data presentation (see line 345).

Findings: This section seems appropriate.

Discussion and conclusion:
This section needs more explanation regarding the relationship between research variables.

We agree with this comment. We have added the following to line 549: “*This could explain the correlations observed between learning in the SRTT task and in the sign language task, as well as between the SRTT and the knot-tying task. These tasks share the common requirement of “chunk” formation,* *allowing participants to perform the motor sequence more quickly and accuracy with repetition. In contrast, SA relies on mechanisms aimed at reducing sensory prediction errors (6,60). This may account for the links found between the Jump task and the Filling Cups without Spilling task, as well as between the Jump task and the Mirror Drawing task, as these tasks require sensory error correction, enabling participants to correct the error more quickly and accuracy with repetition*”.

6. PLOS authors have the option to publish the peer review history of their article ([what does this mean?](https://c05y1x9s.r.us-east-2.awstrack.me/L0/https:%2F%2Fjournals.plos.org%2Fplosone%2Fs%2Feditorial-and-peer-review-process%23loc-peer-review-history/1/010f0191ba8a5740-a1b30736-8268-43e8-a18e-326055cf9d18-000000/SD0FYL2fmYJhSFLWd0-4Zmhkcm8=175)). If published, this will include your full peer review and any attached files.


If you choose “no”, your identity will remain anonymous but your review may still be made public.


**Do you want your identity to be public for this peer review?** For information about this choice, including consent withdrawal, please see our [Privacy Policy](https://c05y1x9s.r.us-east-2.awstrack.me/L0/https:%2F%2Fwww.plos.org%2Fprivacy-policy/1/010f0191ba8a5740-a1b30736-8268-43e8-a18e-326055cf9d18-000000/_MiEmyG4xNQk0xmxRTV_MASColM=175).

Reviewer #1: No

Reviewer #2: No
