# Supplementary material for: From lab to real life: Is there a link between lab-based and ecological assessment of Procedural Perceptual-Motor Learning tasks?
Source: PLoS One. 2025 Apr 7;20(4):e0319715. doi: 10.1371/journal.pone.0319715 (PMC11975140; doi:10.1371/journal.pone.0319715)
Supplement: Attachment — Submitted filename: Response to reviewer2.docx [file pone.0319715.s004.docx]

**Review Comments to the Author**

Please use the space provided to explain your answers to the questions above. You may also include additional comments for the author, including concerns about dual publication, research ethics, or publication ethics. (Please upload your review as an attachment if it exceeds 20,000 characters)

Reviewer #1: The authors have made some revisions to the manuscript; however, the images are of low resolution and unclear. They need to be improved to ensure that readers can accurately interpret them.

The PDF version of the manuscript currently displays lower-resolution images for convenience during the review process. However, high-resolution versions of these images are available and can be accessed by clicking on them. If the manuscript is accepted, the high-resolution images will be included in the final publication.

Reviewer #2: HI.

The article titled " From lab to real life: Is there a link between lab-based and ecological assessment of procedural perceptual-motor learning tasks? " seems to be a valuable and practical PAPER, but it needs a major revision.
Dear authors, please check and respond to the following:
In general, the number of words in the article is very large and needs to be shortened and summarized.

We have carefully revised the manuscript to improve clarity and conciseness. To preserve important details, we propose moving the description of the ecological tasks to the supplementary materials. This would reduce the word count from 8,282 to 7,605.

1. Abstract:

The exact amount of F, P, χ², ...should be provided.

Thank you for your comment. We have added this information to the abstract.

2. Keywords:

All keywords should be based on the mesh (Medical Subject Headings).

Thank you for pointing this out. There are few MeSH terms related to our topic. We included ‘Implicit Learning’ and ‘Motor Control’ as MeSH terms, but also kept the other keywords to maintain precision and capture the full scope of our study (line 56).

3. Introduction:

Despite the practicality of the present article, the authors have not correctly stated the importance of the necessity of the research.

The relationship between the research variables has not been properly observed.

Thank you for your valuable feedback. While lab-based tasks are commonly used to study motor learning, their relevance to real-world skill acquisition remains uncertain (see 7 and 42). This uncertainty presents a significant gap in the field, as it raises important questions about the ecological validity of traditional motor learning research. If lab-based findings do not accurately reflect real-world conditions, this could limit the applicability of decades of research to practical settings, such as sports and rehabilitation. In response, we have revised the introduction to clearly articulate the importance and necessity of the research. Specifically, we have emphasized the practical implications of bridging the gap between lab-based and ecological tasks and clarified the relationships between the research variables (Lines 125, 133 and, 150).

4. Method:

Has a code of ethics been adopted for this research?

Thank you for your question. The research protocol was reviewed and approved by our institutional Research Ethics Committee (CER 2020-320, Comité d’Ethique et de Recherche de l’Université Fédérale de Toulouse), ensuring that the study adhered to ethical standards. However, as this committee does not issue IRB numbers, we are unable to provide one. Nonetheless, the study was conducted in full compliance with ethical guidelines (see line 161).

This section has been written very long.

We have shortened this section while ensuring that all elements essential for the reader's understanding are retained. For the sake of transparency and reproducibility of results, we have included all methodological details but have moved some of the less critical information to the supplementary data (highlighted in green in the manuscript, lines 248 to 285).

On what basis was the number of research samples selected? Has G-power been considered?

Thank you for your comment regarding the selection of our sample size and the consideration of statistical power. The relatively small sample size has been mentioned as a limitation of the study in our discussion (line 675). However, our sample is comparable to those used in similar studies investigating perceptual-motor procedural learning, as seen in one of our previous studies (Martin et al., 2023; <https://doi.org/10.1007/s10072-023-06724-w>). In this review, we intentionally reported effect sizes to ensure transparency, as they provide a robust way to assess the magnitude of effects independently of the p-value, reducing reliance on statistical power for result interpretation. Post hoc analyses using G-Power indicate that with observed rho values ranging from 0.265 to 0.395, the statistical power of the study lies between 0.52 and 0.84. A common belief is that statistical power must always be at least 80% or higher. While this may be a reasonable expectation for clinical studies where significant risks or inconveniences for patients are involved, it is not always necessary in other contexts. For example, in laboratory studies or retrospective correlation analyses, a lower power may be acceptable depending on the research goals and constraints (Dorey et al., 2011; <https://doi.org/10.1007/s11999-010-1435-0>).

5. Discussion and Conclusion:

The explanation of the findings in the first part of the discussion requires general editing.
It seems very necessary to present and compare the results of this research with the results of consistent and inconsistent research.

Thank you for your feedback. We agree that the first part of the discussion could benefit from further editing to enhance clarity and better integrate of our findings with the existing literature. We have proposed revisions to address your concerns.

6. References:
Authors are advised to use up-to-date references.

We have reviewed our references and added the following:

Wu S, Éltető N, Dasgupta I, Schulz E. Chunking as a rational solution to the speed–accuracy trade-off in a serial reaction time task. *Sci Rep*. 2023 May 11;13(1):7680.

Tosatto L, Fagot J, Nemeth D, Rey A. The Evolution of Chunks in Sequence Learning. *Cognitive Science*. 2022;46(4):e13124.

Tosatto L, Fagot J, Nemeth D, Rey A. Chunking as a function of sequence length. *Anim Cogn*. 2024; 1:13.

Conway CM. How does the brain learn environmental structure? Ten core principles for understanding the neurocognitive mechanisms of statistical learning. *Neuroscience & Biobehavioral Reviews*. 2020 May;112:279–99.

Haar S, Sundar G, Faisal AA. Embodied virtual reality for the study of real-world motor learning. *PLOS ONE*. 2021 Jan 27;16(1):e0245717.

Worschech F, Passarotto E, Losch H, Oku T, Lee A, Altenmüller E. What Does It Take to Play the Piano? Cognito-Motor Functions Underlying Motor Learning in Older Adults. *Brain Sci*. 2024 Apr 20;14(4):405.
